# Night Duty and the Patient's Sufferings Increasing Terrors of Each Night

**Published:** 1918-12-21

**Authors:** Paul Pry


### A PATIENT'S PROTEST.

### Night Duty and the Patient's Sufferings.

### Increasing Terrors of Each Night.

*To the Editor of* THE HOSPITAL.

SIR,—I would like to see the subject of Nurse Dale's letter very thoroughly taken up. I, as another nurse, who has also been a patient, have often marvelled at the strange absence of intelligence about the management of night duty in hospitals. It would be nice if it could be reformed out of the system by ourselves, but if it cannot I am among those who would be glad to have public opinion reform it out of us.

We teach our probationers—the village girl old enough to attend lectures on home nursing is taught—the importance of sleep in illness, and then we send them—the probationers, anyhow—on night duty with so much to do that they have to keep patients awake nearly all night to get through.

Four A.M. for starting washings is really a moderate statement of what happens. It is as often earlier as later. As I once heard a patient in hospital say, "When I was here before it was 4.30; it isn't even four now. I wonder what time it will have got to if I ever come again." And the ward wit replied, "The patients, I expect, will be on night duty."

Surely there are some hospitals where they have found a way for keeping such obviously day duties *to* the day, though *I* don't know of one. Would not some matrons or nurses who can manage that tell the others *how*?

PAUL FRY.

